# A case of intrathyroid parathyroid tumor that was difficult to diagnose by ultrasonography

**DOI:** 10.1186/s13089-020-00164-9

**Published:** 2020-04-03

**Authors:** Nobuyuki Takemoto, Ai Koyanagi, Masanori Yasuda, Yuya Yamamoto, Hiroshi Yamamoto

**Affiliations:** 1 Department of Breast & Endocrine Surgery, Japan Medical Alliance East Saitama General Hospital, 5-517, Yoshino, Saitama-Pref, Satte, 340-0153 Japan; 2 Research and Development Unit, Parc Sanitari Sant Joan de Déu, Fundació Sant Joan de Déu, CIBERSAM, Sant Boi de Llobregat, Barcelona, Spain; 3 ICREA, Pg. Lluis Companys 23, Barcelona, Spain; 4 Department of Pathology, Saitama Medical University International Medical Center, 1397-1, Yamane, Hidaka, Saitama-Pref Japan; 5 Department of Endocrine Internal Medicine, Japan Medical Alliance East Saitama General Hospital, 5-517, Yoshino, Satte, Saitama-Pref Japan; 6 Geriatric Health Service Facility (COSMOS), Japan Medical Alliance Yokohama Stroke and Brain Center, 1-2-1 Takigashira, Isogoku, Yokohama, Kanagawa-Pref Japan

**Keywords:** Intrathyroid parathyroid adenoma, Ultrasonography, ^99m^Tc-MIBI, Difficult-to-diagnose

## Abstract

**Background:**

With advances in diagnostic imaging such as ultrasonography (US), computed tomography (CT), and ^99m^Tc-MIBI-sestamibi (MIBI) scintigraphy, localized diagnosis of hyperparathyroidism (pHPT) has become possible with considerable accuracy. However, even with the use of these imaging techniques, since intrathyroid parathyroid tumors exist as a mass within the thyroid, it is often difficult to distinguish from thyroid masses. Although there have been various reports on US images of intraparathyroid tumors, we experienced a case with US images that were distinct from previous reports. Herein we present a case of an intrathyroid parathyroid adenoma (IPA) that was difficult to diagnose, with a main focus on US images.

**Case presentation:**

A 53-year-old man with a diagnosis of hyperparathyroidism was referred to our department in December 2018. Ultrasonography revealed a tumor that was located in the inferior pole of the right lobe of the thyroid gland and no parathyroid mass was observed. The tumor had an irregular round shape and showed heterogeneous hyperechogenicity with a defined margin, but within it, there were a few irregular and hypoechogenic area with unclear margins, while the tumor had a mosaic appearance at first glance. Although ^99m^Tc-MIBI scintigraphy showed accumulation at the same location in delayed phase, it was difficult to determine the presence of a parathyroid tumor on the image. The patient underwent an operation on April 2019 and the tumor could not be identified on both naked eye and palpation. We used US intraoperatively to define the location and resected the tumor. A parathyroid adenoma was diagnosed by frozen section and the final diagnosis was an intrathyroid parathyroid adenoma.

**Conclusion:**

We experienced an IPA presenting an US image that was atypical and has previously not been reported. IPA has no established US image to confirm the diagnosis and even with the use of other imaging techniques, a definitive diagnosis often cannot be established. Thus, our recommendation based on the current situation is that operation with intraoperative diagnosis using frozen section should be conducted if hypercalcemia and high I-PTH are observed and when localization sites in MIBI and US coincide.

## Introduction

The basis of treatment of primary hyperparathyroidism (pHPT) is parathyroidectomy of the responsible lesion, and thus, preoperative localization is crucial. With advances in diagnostic imaging such as ultrasonography (US), computed tomography (CT), and ^99m^Tc-MIBI-sestamibi (MIBI) scintigraphy, localized diagnosis of pHPT has become possible with considerable accuracy [1]. However, even with the use of these imaging techniques, since intrathyroid parathyroid tumors exist as a mass within the thyroid, it is often difficult to distinguish from thyroid masses. Although there have been various reports [1–3] on US images of intraparathyroid tumors, we experienced a case with US images that were distinct from previous reports. Herein we present a case of an intrathyroid parathyroid adenoma (IPA) that was difficult to diagnose, with a main focus on US images.

## Case presentation

**Fig. 1:**
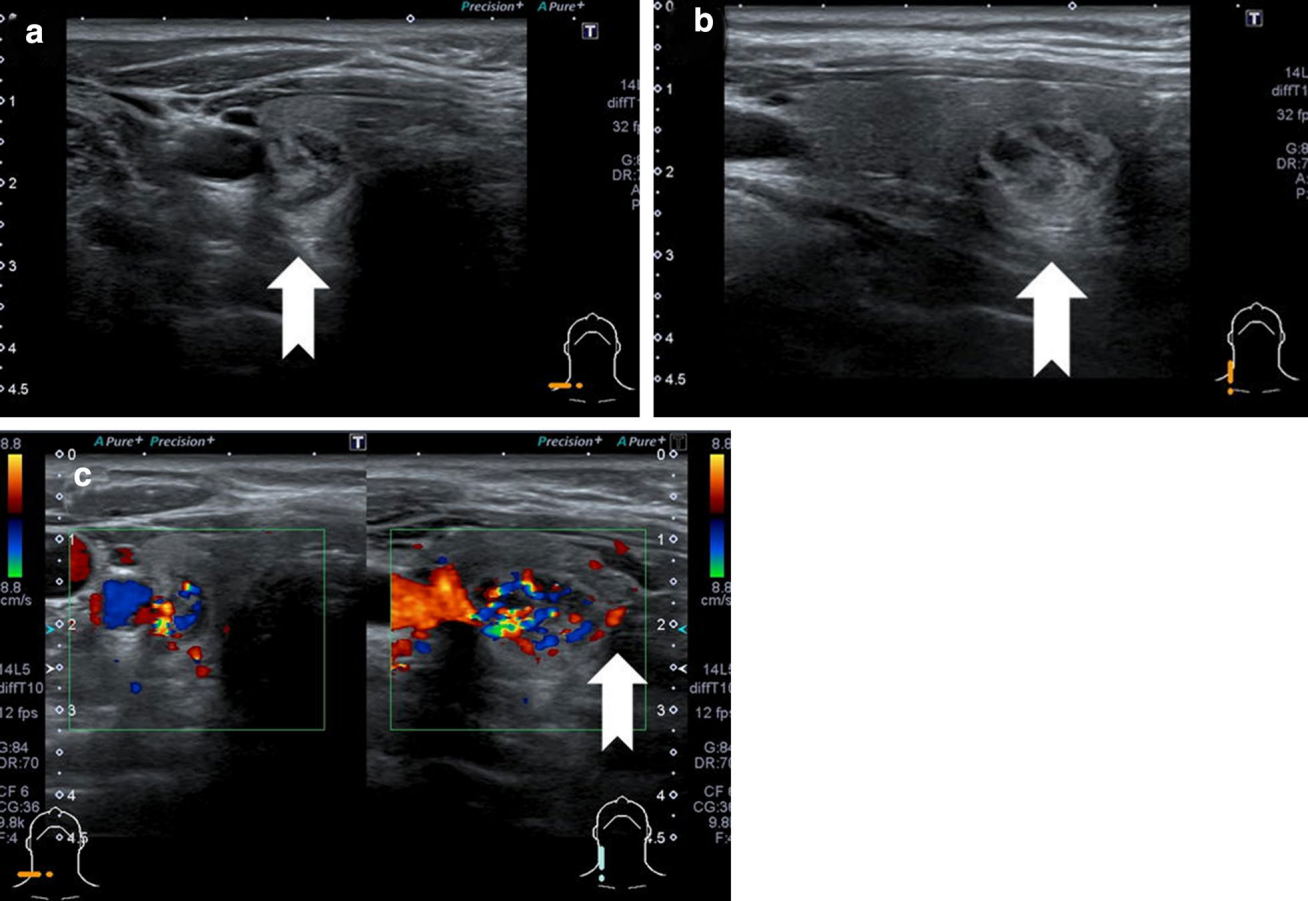
Ultrasonographic imaging. **a** Transverse image. **b** Longitudinal image. **c** Color Doppler imaging. The tumor ((**a, b**) arrow) was located in the inferior pole of the right lobe of the thyroid gland and no parathyroid mass was observed. The size of the tumor was 18 × 13 × 11 mm (anteroposterior × transverse × craniocaudal dimension). The tumor had an irregular round shape and showed heterogeneous hyperechogenicity with a defined margin, but within it, there were a few unclear margins, irregular and hypoechogenic area, and the tumor had a mosaic appearance at first glance. Color Doppler sonography showed that the blood vessels ((**c**) arrow) flowing into the tumor were thick and marked blood flow was also observed within the tumor

A 53-year-old man with a diagnosis of hyperparathyroidism was referred to our department in December 2018. The results of laboratory examinations were compatible with hyperparathyroidism: intact-PTH 94 pg/ml (normal range 10–65 pg/ml), serum Ca 11.9 mg/dl (normal range 8.2–10.4 mg/dl), and serum P 2.4 mg/dl (normal range 2.5–4.6 mg/dl). Thyroid function tests (TSH, Free-T3, Free-T4) were all within normal limits. Cervical tumors and lymphadenopathy were not identified on palpation. The patient showed no signs of hoarseness, dysphagia and dysphonia. His past medical history included ureter stones and type 2 diabetes, and he had no kidney diseases that can cause secondary hyperparathyroidism. There were no notable family medical histories. US was performed with a 7.5-MHz linear transducer (TOSHIBA MEDICAL SYSTEMS; SSA-790A, TOCHIGI, JAPAN). A tumor was found in the inferior pole of the right lobe of the thyroid gland and no parathyroid mass was observed. The tumor had an irregular round shape and showed heterogeneous hyperechogenicity with a defined margin, but within it, there were a few unclear margins, irregular and hypoechogenic area, and the tumor had a mosaic appearance at first glance (Fig. 1a, b). The size of the tumor was 18 × 13 × 11 mm (anteroposterior × transverse × craniocaudal dimension). Color Doppler sonography showed that the blood vessels flowing into the tumor were thick and marked blood flow was observed within the tumor (Fig. 1c). No lymph node swelling was observed in the cervical regions.

**Fig. 2:**
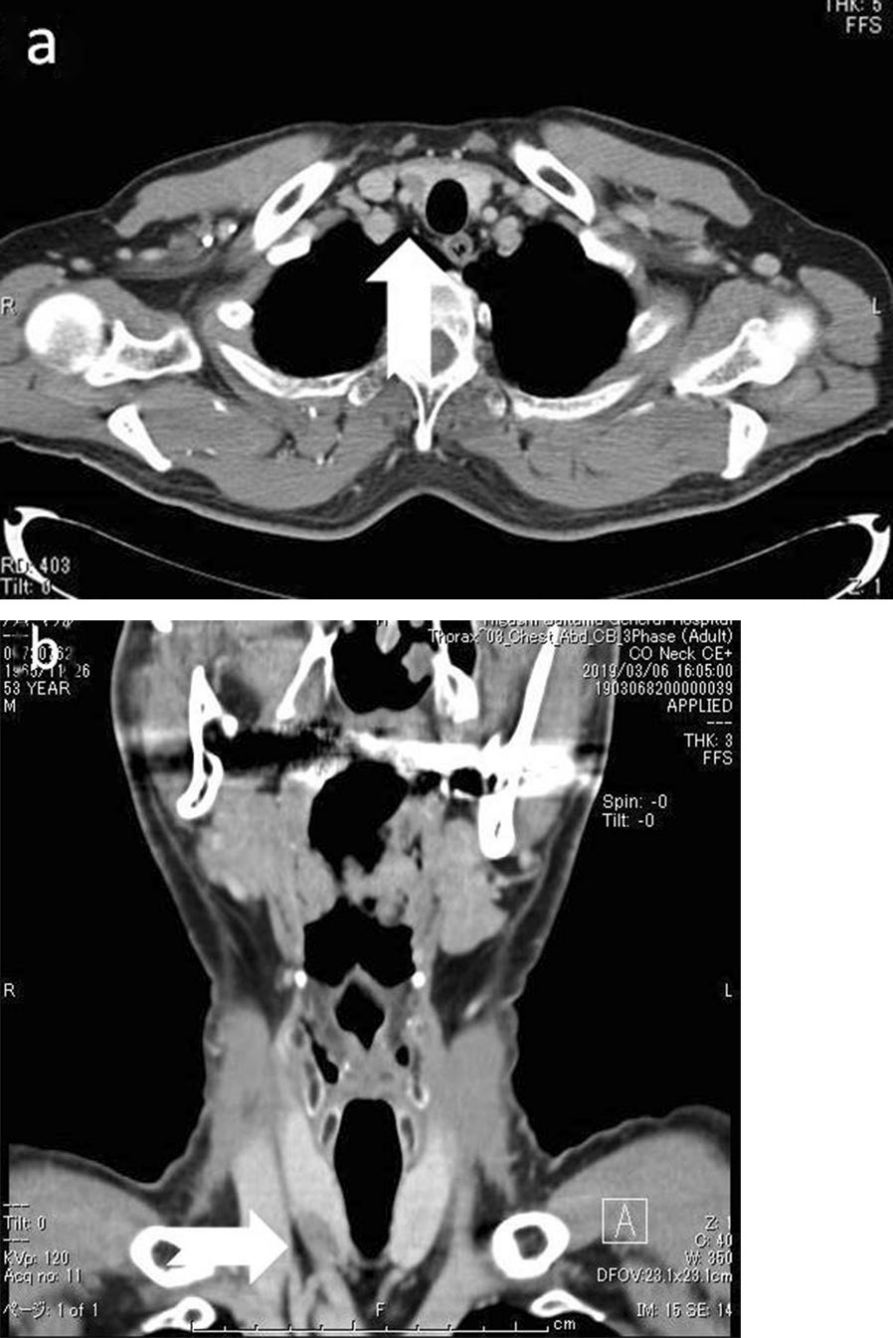
Enhanced computed tomography. **a** Transverse image. **b** Sagittal image. Images obtained by CT revealed a low density mass which was slightly enhanced by contrast injection in the right thyroid lobe

**Fig. 3:**
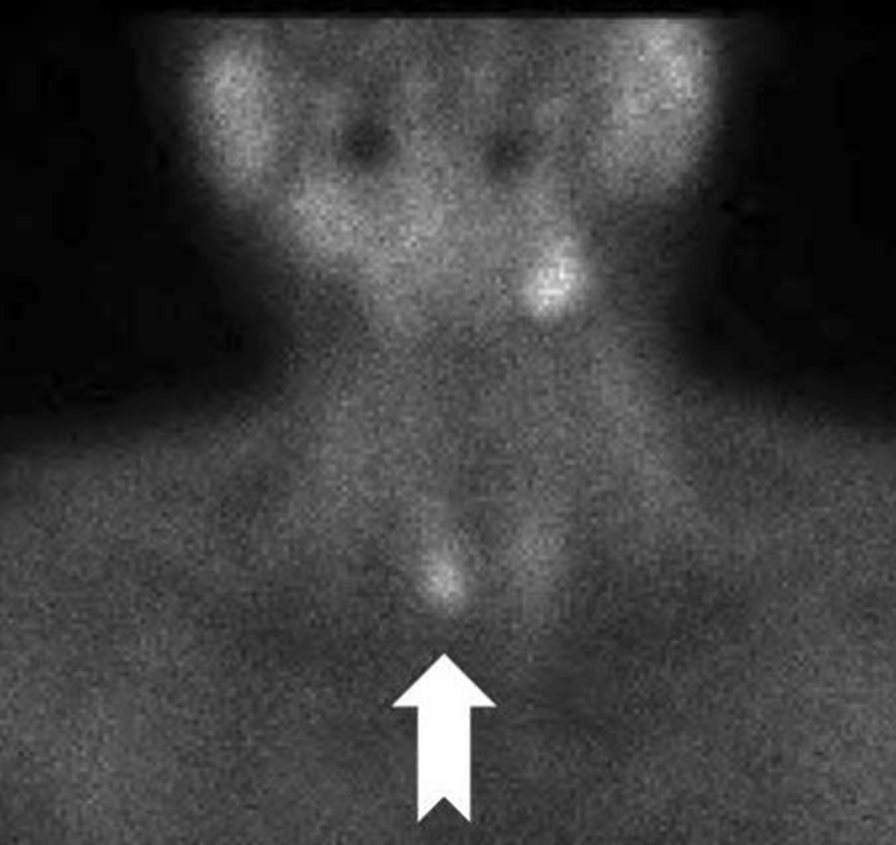
MIBI scintigraphy The MIBI scintigraphy showed accumulation near the lower right pole and strong accumulation even in the delayed phase, suggestive of a parathyroid adenoma

Images obtained by CT revealed a low density mass which was slightly enhanced by contrast injection in the right thyroid lobe (Fig. 2a, b). No other abnormality was identified on thoraco-abdominal CT. The MIBI scintigraphy showed accumulation near the lower right pole and strong accumulation even in the delayed phase, suggesting parathyroid adenoma (Fig. 3). The image was very atypical for a parathyroid adenoma, and the possibility of hyperplasia and cancer was also considered. Considering the risk of dissemination, we decided not to carry out fine needle aspiration cytology (FNAC) but to attempt intraoperative diagnosis using frozen section and to treat at the same time.

**Fig. 4:**
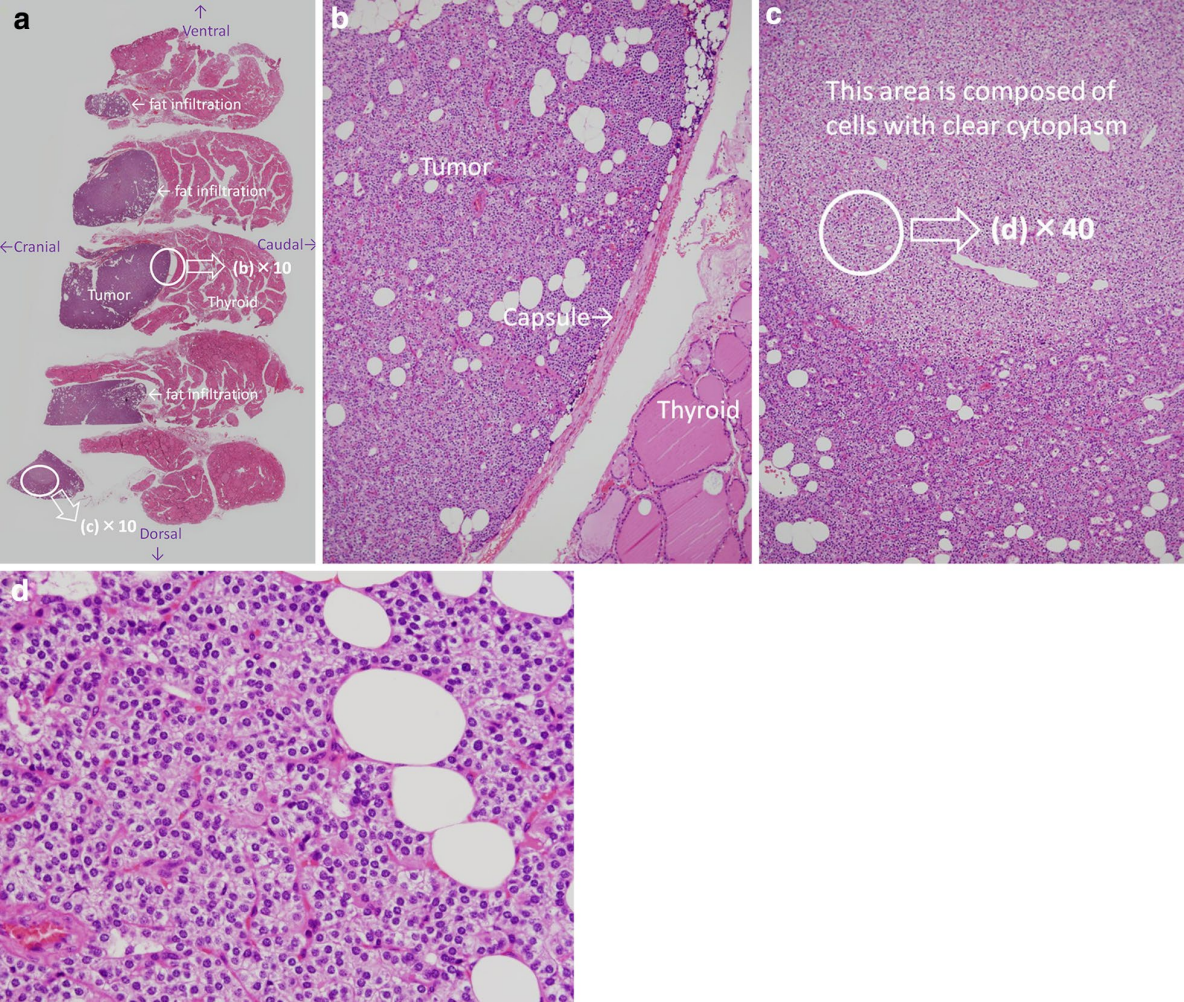
Pathological findings (Hematoxylin and Eosin stain) **a** Semi-macroscopic view (loupe image). The longitudinal images of the tumor are arranged in order. **b** × 10. The parathyroid adenoma was surrounded by a thin fibrotic capsule. **c** × 10. There were some nodular areas, which are composed of cells with clear cytoplasm, and there was scattered fatty infiltration. **d** × 40. The parathyroid adenoma showed no variant type and was almost uniform throughout. There was no evidence of malignancy

The patient underwent resection of the tumor under collar incision on April 2019. As the tumor could not be identified on both naked eye and palpation, we used US intraoperatively and confirmed that the tumor was located in the inferior pole of the right lobe of the thyroid gland. The cranial side of the tumor was identified, from which the thyroid was incised and a right partial lobectomy was performed. Pathological examination revealed a parathyroid adenoma that was surrounded by a thin capsule and showed no variant type (Fig. 4b, d). However, there were some nodular areas composed of cells with clear cytoplasm (Fig. 4c), and there was scattered fatty infiltration. There was no evidence of malignancy. The final diagnosis was IPA. The patient recovered well after surgery and was discharged on the 5th day. Postoperatively, serum calcium and I-PTH were rapidly normalized, and no increase has been observed as of July 2019.

## Discussion

The parathyroid glands can become misaligned during development [2]. The vast majority of these displaced parathyroid glands are found in the inferior gland (90%) and those in the superior gland are rare [2, 4, 5]. This is because the developmental origin of parathyroid glands differs between the superior and inferior glands [2]. The superior parathyroid glands are derived from the fourth pharyngeal pouch and lie on the dorsal surface of the thyroid gland, while the inferior parathyroid glands originate from the third pharyngeal pouch accompanying the thymus, and are in a more inferior position than the superior parathyroid glands derived from the fourth pharyngeal pouch [6]. Therefore, the inferior glands are more likely to become misaligned due to more migration of embryological tissue.

Ectopic parathyroid gland is understood to be caused by the development of adenoma or hyperplasia from parathyroid glands that have been displaced, such as in the thyroid or in the thymus [2, 7]. Among pHPT, IPA is rare with its prevalence reported to be between 1.4 and 2.1% [3, 8]. IPA is more frequent on the right lobe [2–4], and is divided into complete and partial types in encircled form, but the frequency of these types are reported to be the same [2, 5, 9].

Cervical US, contrast-enhanced CT, and MIBI scintigram are useful for the localization of parathyroid tumors. The rate of correct diagnosis is very high especially when the localization in cervical US and MIBI scintigram coincide, and at present, the combination of US and 99mTc-MIBI SPECT/CT is most recommended [1]. However, in the case of IPA, the interpretation of imaging findings is difficult compared to pHPT cases that exist in the normal position [2].

When normal parathyroid tumor is observed in US, it is shown as a mass showing flat, elliptical, clear borderline, internal homogeneous hypoechoic mass on the dorsal side of the thyroid capsule depicted in high echo level, and in the color Doppler method, blood flow inside the mass is abundantly observed [1]. The previously reported US findings in IPA include the following: (a) the tumor is not flat but has an oval to near-spherical shape because the compression from the cervical spine and thyroid is weak [2]; (b) it often has the same properties as parathyroid tumor [2]; (c) it may be confused with cysts, but identification is possible by recognizing blood flow signals inside [1]; (d) Yubata [3] reported that the typical sonographic appearance of IPA consists of a regular shape, smooth border, hypoechoic level of internal echo, solid content, a hyperechoic line on the ventral surface of the parathyroid gland, and the presence of feeding vessels. In particular, a hyperechoic line on the ventral surface of the parathyroid gland has been reported as a finding characteristic of IPA. However, only 20% of reported cases had this hyperechoic line [2], and its diagnostic utility has not been determined.

In the US image of our case, the border of the mass was relatively clear, and the internal echo was homogeneous and hyperechoic, but within the mass, there were several irregularly shaped, unclear borders and hypoechoic areas, which resembled a mosaic at first glance. In the US transverse image, the hyperechoic line reported by Yubata et al. [3] appeared to be present on the ventral side of the adenoma, but the longitudinal image showed that the line was present almost all around the mass. The line was not ventrally specific, and is likely to have been the capsule of the mass. These findings were not specific enough to provide a definitive diagnosis of IPA based on US only.

There are several possible reasons for the atypical US image found in our case. First, Fig. 4a is a pathological picture in which the longitudinal images of the mass are arranged in order, but notice that the shape of the mass is different from the US image (Fig. 1b). However, when the US Probe was placed on the mass and compression was applied, the mass fell to the cranial side, and the image in Fig. 1b was obtained. The fat concentrated on the thyroid side (= caudal side) of the mass moved to the ventral side, so this may have been depicted as irregular hypoechoic (i.e., mosaic-like findings).

Also, when the pathological picture is reviewed, there is a nodular area in one part of the mass, and the area consists of clear cytoplasm in the surrounding area. Although speculative, since the US visualizes a mass as a shadow, factors such as density of cells, water content of each cell, and inherent properties of US such as scattering, absorption, attenuation, refraction, reflection, etc. may have interacted to create this image.

Next, MIBI is an essential modality for diagnosis of pHPT due to its high specificity, but may give rise to false-negatives for small parathyroid glands smaller than 300 mg [1]. In addition, pHPT is likely to be associated with various nodular thyroid lesions [10, 11], and MIBI is known to be frequently accumulated in thyroid-derived nodules and to give rise to false-positives [1, 3]. However, thyroid tumors tend to be excreted in the delayed phase because they wash out faster than the parathyroid gland, and there is also a report that differentiation is possible to some extent [12]. In this case, strong accumulation was observed even in the delayed phase, and retrospective observation suggests that it was more likely to be IPA rather than a goiter.

In addition, CT has recently been used to provide diagnosis for enlarged parathyroid glands by contrast pattern [13], but in the case of IPA, it is strongly affected by contrast enhancement of the background thyroid, so it is very difficult to confirm the tumor contrast pattern. Although the existence of a tumor can be confirmed by CT, currently, definitive diagnosis is difficult to establish.

Recently, it has been reported that a single-photon emission computed tomography/computed tomography (SPECT/CT) imaging is useful for localization of parathyroid lesions and identification of the size of adenomas or hyperplastic glands [14, 15]. SPECT/CT has been reported to be capable of detecting parathyroid adenomas even with a greatest axial diameter of 0.6 cm [14]. In Japan, this equipment is still not widely available; however, workstations to create a fusion image of MIBI SPECT and CT are being used in some facilities without an exclusive SPECT/CT device [16]. There is a growing expectation for wider availability of this method.

Some authors have suggested that it may be possible to determine whether a tumor is of parathyroid origin or not by measuring intact-PTH of the lavage fluid from fine needle aspiration of the lesion [17]. However, in facilities where the number of cases is limited, this may not be feasible in terms of cost. Furthermore, given that the parathyroid tissue is a tissue that can easily engraft [18] and disseminate [2], while puncture can cause bleeding, this method may not be practical especially for cases where parathyroid cancer is suspected. Indeed, FNAC is contraindicated for parathyroid cancer [19], and even if IPA is suspected clinically, performing FNAC carries a risk as preoperative diagnosis of parathyroid cancer is difficult.

At present, it is difficult to make a definitive diagnosis of IPA before operation, although the accuracy of diagnosis of IPA can be increased by combining various imaging techniques. In particular, there are no typical images that are specific enough to establish a definitive diagnosis with US. In the case of suspected IPA, currently, the optimal measure may be to perform resection together with intraoperative US and to confirm parathyroid tissue with frozen section. Development of new diagnostic imaging techniques is warranted for preoperative diagnosis of IPA.

## Conclusion

We experienced an IPA presenting an US image that was atypical and has previously not been reported. IPA has no established US image to confirm the diagnosis and even with the use of other imaging techniques, a definitive diagnosis often cannot be established. Thus, our recommendation based on the current situation is that operation with intraoperative diagnosis using frozen section should be conducted if hypercalcemia and high I-PTH are observed and when localization sites in MIBI and US coincide.

## Data Availability

This manuscript adhered to Hospital policies regarding image collection and research. Data can be made available upon request.

## Abbreviations

US: Ultrasonography
CT: Computed tomography
MIBI: ^99m^Tc-MIBI-sestamibi
pHPT: Hyperparathyroidism
IPA: Intrathyroid parathyroid adenoma
FNAC: Fine needle aspiration cytology
